# Ultrasound experiments on acoustical activity in chiral mechanical metamaterials

**DOI:** 10.1038/s41467-019-11366-8

**Published:** 2019-07-29

**Authors:** Tobias Frenzel, Julian Köpfler, Erik Jung, Muamer Kadic, Martin Wegener

**Affiliations:** 1 Institute of Applied Physics, Karlsruhe Institute of Technology, 76128 Karlsruhe, Germany; 2 Institute of Nanotechnology, Karlsruhe Institute of Technology, 76021 Karlsruhe, Germany; 3 Institut FEMTO-ST, CNRS, Université de Bourgogne Franche-Comté, 25000 Besançon, France

**Keywords:** Mechanical properties, Metamaterials, Acoustics

## Abstract

Optical activity requires chirality and is a paradigm for chirality. Here, we present experiments on its mechanical counterpart, acoustical activity. The notion “activity” refers the rotation of the linear polarization axis of a transversely polarized (optical or mechanical) wave. The rotation angle is proportional to the propagation distance and does not depend on the orientation of the incident linear polarization. This kind of reciprocal polarization rotation is distinct from nonreciprocal Faraday rotation, which requires broken time-inversion symmetry. In our experiments, we spatiotemporally resolve the motion of three-dimensional chiral microstructured polymer metamaterials, with nanometer precision and under time-harmonic excitation at ultrasound frequencies in the range from 20 to 180 kHz. We demonstrate polarization rotations as large as 22° per unit cell. These experiments pave the road for molding the polarization and direction of elastic waves in three dimensions by micropolar mechanical metamaterials.

## Abstract (web summary)

Here, the authors report an effect analogous to optical activity in a 3D mechanical micro-lattice composed of chiral unit cells. They spatiotemporally resolve the motion of metamaterial beams at ultrasonic frequencies with nanometric precision and show up to 22° polarization rotation per unit cell.

## Introduction

An object is geometrically chiral if it lacks centrosymmetry, mirror symmetries, and rotation-reflection symmetries^1–3^. Chirality, or handedness, has played an important role in physics, optics, chemistry, biology, and medicine for decades already. Examples are optical activity^4–7^ and circular dichroism^8,9^, exclusively right-handed DNA double-helix strands^10^, exclusively left-handed neutrinos^11^, chiral topological fermions^12^, stereochemistry^13^, and the effect of drugs depending on the handedness of the underlying molecules^14^.

Surprisingly, chirality did not play a major role in the context of mechanics for many years. This has started to change recently. For example, geometrical chirality is important in plant seedpod mechanics^15^. Chirality has enabled topological effects at finite frequencies^16,17^, and has opened degrees of freedom beyond Cauchy elasticity in the stationary regime^18,19^. Handedness in shearing auxetics has created rigid and compliant structures^20^. The reviews^21–25^ provide an overview on the status of the field of mechanical metamaterials in general. Chiral phonons in quasi two-dimensional monolayers of noncentrosymmetric tungsten diselenide crystals have only recently been inferred from optical spectroscopy^26^. However, one of the paradigms of chirality, namely “activity” has so far only been a theoretical possibility for generalized elastic continua^27–29^.

In the presence of optical activity^4,7^ or acoustical activity^27–29^, an incident transverse linear polarization of a propagating wave rotates: the eigenpolarizations of the chiral medium are not linear but rather circular, with a lifted degeneracy between left- and right-handed circular modes. Therefore, a linear incident polarization must be decomposed into the two circular-polarization eigenmodes, which propagate with different phase velocities. The resulting phase difference accumulated during propagation leads to a continuous rotation of the linear polarization axis versus the propagation coordinate. Optical and acoustical activity therefore allow, for example, to convert an incident linear polarization into the orthogonal one. Both are reciprocal effects, hence distinct from the nonreciprocal Faraday effect, which requires broken time-reversal symmetry, yet does not need chirality^7^. Regarding mechanics, yet a different kind of mode conversion has recently been observed in two-dimensional centrosymmetric metamaterials^30^. There, the conversion between in-plane longitudinal (compression) and in-plane transverse (shear) modes did not require chirality.

In this Letter, we report the experimental observation of the phenomenon of acoustical activity in three-dimensional (3D) chiral polymer metamaterials by direct spatiotemporal imaging at ultrasound frequencies, using cross-correlations of optical microscopy images taken under synchronized stroboscopic illumination. The experimental results agree well with numerical phonon band-structure calculations and simulations for the considered experimental geometry using finite-size samples.

## Results and discussion

### Beyond Cauchy elasticity

Let us start by recalling that effects of chirality are neglected in mechanics on the level of textbook linear Cauchy elasticity^31^. Plainly speaking, Cauchy continuum mechanics can be seen as “point mechanics”^32–34^. Therefore, Cauchy elasticity becomes exact if the wavelength of an elastic wave, *λ*, is much larger than the crystal lattice constant, *a*, and if the sample size, *L*, is much larger than *a*, too. Therefore, at least one of the two conditions, *λ*/*a*≫1 and *L*/*a*≫1, needs to be violated to observe acoustical activity. Violating both conditions simultaneously is expected to yield larger effects. This means that we need to investigate the phonon band structure in the middle between the Γ-point and the Brillouin-zone edge (by symmetry the bands are degenerate at the Brillouin-zone edge) for beams of finite and infinite lateral extent *L*_*x*_ = *L*_*y*_, with the number of unit cells *N*_*x*_ = *N*_*y*_ = *L*_*x*_/*a* = *L*_*y*_/*a* in the two directions perpendicular to the wave propagation along *z*.

### Band-structure analysis

**Fig. 1:**
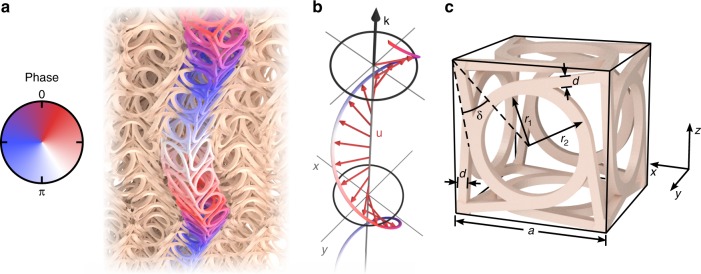
Chiral phonon eigenmode and unit cell. **a** Snapshot of a calculated chiral phonon eigenmode with eigenfrequency *ω*_1_ propagating along the *z*-direction in an infinitely extended 3D chiral micropolar metamaterial crystal. For clarity, all unit cells except for one column along the propagation axis (*z*-axis) are shown semitransparent and the displacements are largely exaggerated. **b** Simplified representation only showing the behavior of the unit cells’ centers of mass, forming a helix that is moving along the *z*-axis versus time. An animation of **a** and **b** is shown in Supplementary Video 1. **c** Single-unit cell (cf. ref. ^19^). Geometrical parameters are cubic lattice constant *a* = 250 μm, *d*/*a* = 0.06, *r*_1_/*a* = 0.32, *r*_2_/*a* = 0.4, and *δ* = 34.8°. For the bulk constituent polymer material we have chosen the Young’s modulus *E* = 4.18 GPa, mass density *ρ* = 1.15 g cm^−3^, and Poisson’s ratio *ν* = 0.4. The wave number is *k*_*z*_ = *π*/(4*a*) and the angular frequency is *ω*_1_ = 2*π* × 107 kHz, equivalent to *a*/*λ*_0_ = 0.0095, with the wavelength *λ*_0_ of pressure waves in the bulk constituent polymer material (cf. Fig. 2)

Figure 1a shows a snapshot of a calculated chiral phonon eigenmode (at wave number *k*_*z*_ = *π*/(4*a*), hence angular frequency *ω*_1_≈2*π* × 107 kHz) propagating in an infinitely extended 3D chiral micropolar metamaterial with *N*_*x*_ = *N*_*y*_ = *N*_*z*_→∞ in the linear elastic regime. For clarity, the displacements are largely exaggerated. The behavior is qualitatively similar for other parameters. Panel b exhibits a simplified representation and panel c the underlying chiral metamaterial crystal unit cell (also see ref. ^19^), with geometrical parameters indicated.

**Fig. 2:**
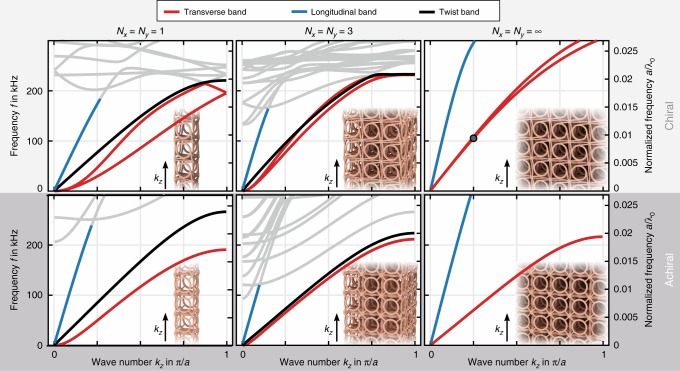
Calculated phonon band structures for chiral metamaterial beams. The left vertical scales are in absolute units, the right scales, *a*/*λ*_0_, are normalized and scalable to other parameters (cf. Fig. 1). We consider wave propagation with wave number *k*_*z*_ in a beam which is infinitely extended along the *z*-direction and which contains *N*_*x*_ × *N*_*y*_ unit cells (with *N*_*x*_ = *N*_*y*_) in the two orthogonal directions. The three panel columns correspond to *N*_*x*_ = *N*_*y*_ = 1, 3, and ∞, respectively. The upper row is for chiral (*δ* = 34.8°) metamaterials beams, the lower row for achiral (*δ* = 0) ones. Transverse or shear-like bands are shown in red (a lifting of degeneracy reflects acoustical activity), pressure-like modes in blue, and twist-like modes in black. The latter are absent for *N*_*x*_ = *N*_*y*_ = ∞. For clarity, higher bands not relevant here are plotted in gray. The insets illustrate the corresponding geometries. The spatial mode depicted in Fig. 1 is highlighted by a circle. Parameters are as in Fig. 1

Figure 2 gives an overview on the theoretical expectations for the corresponding band structures *ω*_*n*_ = *ω*_*n*_(*k*_*z*_) = *ω*_*n*_(−*k*_*z*_), with the angular frequencies *ω*_*n*_ (*n* = 1,2,…) and the wave number *k*_*z*_. All statements concerning the character of the bands made below are based on an investigation of the associated eigenmodes. Here, we assume Bloch-periodic boundary conditions along the *z*-direction and open boundaries of the beams along the *x*- and *y*-direction for finite *N*_*x*_ = *N*_*y*_ (left and center column in Fig. 2). For the limit *N*_*x*_ = *N*_*y*_→∞ (right column in Fig. 2), we assume Bloch-periodic boundary conditions along the *x*- and *y*-direction as well. For a finite square-shaped cross-section *N*_*x*_ × *N*_*y*_, four different bands emerge from the Brillouin-zone center. All other bands (*n* ≥ 5) are of lesser importance here and are hence plotted in gray for clarity. The lowest two modes (*n* = 1,2) shown in red correspond to flexural waves with transverse circular polarization. Here, the center of mass of any given unit cell rotates clockwise or counter-clockwise around the  wave vector **k**, respectively, in circles around its rest position (cf. Fig. 1). In other words, these modes are chiral phonons (upper row of Fig. 2). As usual for thin plates and beams^35^, the flexural modes start from the Γ-point with a parabolic dispersion. The behavior changes toward a linear dispersion if the cross-section of the beam is successively increased.

The absolute and the relative splitting between these two chiral phonon bands tends to zero near the Γ–point and, by symmetry, is also zero at the edge of the first Brillouin zone at |*k*_*z*_| = *π*/*a*. Thus, maximum splitting occurs roughly around the middle of the one-dimensional Brillouin zone, where |*k*_*z*_| = *π*/(2*a*) or *λ*/*a* = 4 with |*k*_*z*_| = 2*π*/*λ*. In analogy to optical activity, for a given frequency *ω* = *ω*_1_ = *ω*_2_, the splitting in wave number, Δ*k*_*z*_, leads to a polarization rotation angle Δ*φ* due to acoustical activity over propagation distance *L*_*z*_ given by Δ*φ* = Δ*k*_*z*_*L*_*z*_/2. The maximum splitting in Fig. 2 decreases with increasing beam cross-section *N*_*x*_ = *N*_*y*_ = *L*_*x*_/*a* = *L*_*y*_/*a*, but remains finite in the limit *N*_*x*_ = *N*_*y*_→∞. This behavior is in line with our above intuitive reasoning starting from Cauchy elasticity as well as with our previous static experiments^19^. In the remainder of this Letter, we will focus on these two chiral phonon bands, i.e., on *n* = 1,2.

Our analysis of phonons based on micropolar cubic-symmetry continua^32^ yields that these two transverse bands live in a subspace orthogonal to the other two bands (*n* = 3,4). This statement is true for wave vectors along the three cubic principal directions, e.g., for the *z*-direction. We, therefore, find several crossings of these bands (rather than avoided crossings). Without chirality, the character of the third (black) and fourth (blue) bands in Fig. 2 would be that of a pure twist and that of a pure compression wave, respectively. In the presence of chirality, their characters are mixed. This band mixing is the direct dynamic counterpart of our recent static experiments on metamaterials with cubic symmetry^19^, in which an axial force imposed onto a beam led to a twist of the beam. In the limit *N*_*x*_ = *N*_*y*_ → ∞, the twist mode (black) is absent because it is disallowed for Bloch-periodic boundary conditions along the *x-*, *y-*, and *z*-directions.

### Connection to acoustical activity

The slopes of all four modes depend on the beam cross-section, hence on *N*_*x*_ = *N*_*y*_. This behavior is the counterpart of the fact that the effective Young’s modulus in the stationary case of micropolar materials is no longer a constant “material parameter”, but rather depends strongly on the geometry, i.e., on the ratio *N*_*x*_ = *N*_*y*_ = *L*_*x*_/*a* = *L*_*y*_/*a*^18^. If we switch off chirality (*δ* = 0 in Fig. 1a) as a control calculation and use otherwise identical geometrical and constituent material parameters (cf. Fig. 1), the behavior becomes ordinary again and the slopes no longer change very much (see lower row in Fig. 2).

For example, on the basis of the data shown for *N*_*x*_ = *N*_*y*_ = 1 in the upper row of Fig. 2, we estimate from the wave number splitting at about |*k*_*z*_| = *π*/(2*a*) an acoustical activity of 17° per unit cell. We have confirmed this estimate by independent numerical calculations for samples with a finite extent along the *z*-direction (see Supplementary Fig. 1). Here, we excite a linearly polarized transverse wave at the bottom of the metamaterial beam. *N*_*z*_ unit cells upwards, a plate with markers allows for monitoring the local displacement vectors versus time. Clearly, the open end of the sample leads to reflections of the elastic waves. However, on the way back, the polarization rotation is reversed due to time-reversal symmetry (unlike for the Faraday effect, see section “Introduction”). Therefore, standing waves along the beam axis do not change the polarization rotation at the top of the beam. Standing waves may well influence the amplitude of the displacements at the top though. As the *z*-axis has fourfold rotational symmetry, the polarization rotation does not depend on the orientation of the incident linear polarization (see Supplementary Fig. 2).

### Measurement setup

**Fig. 3:**
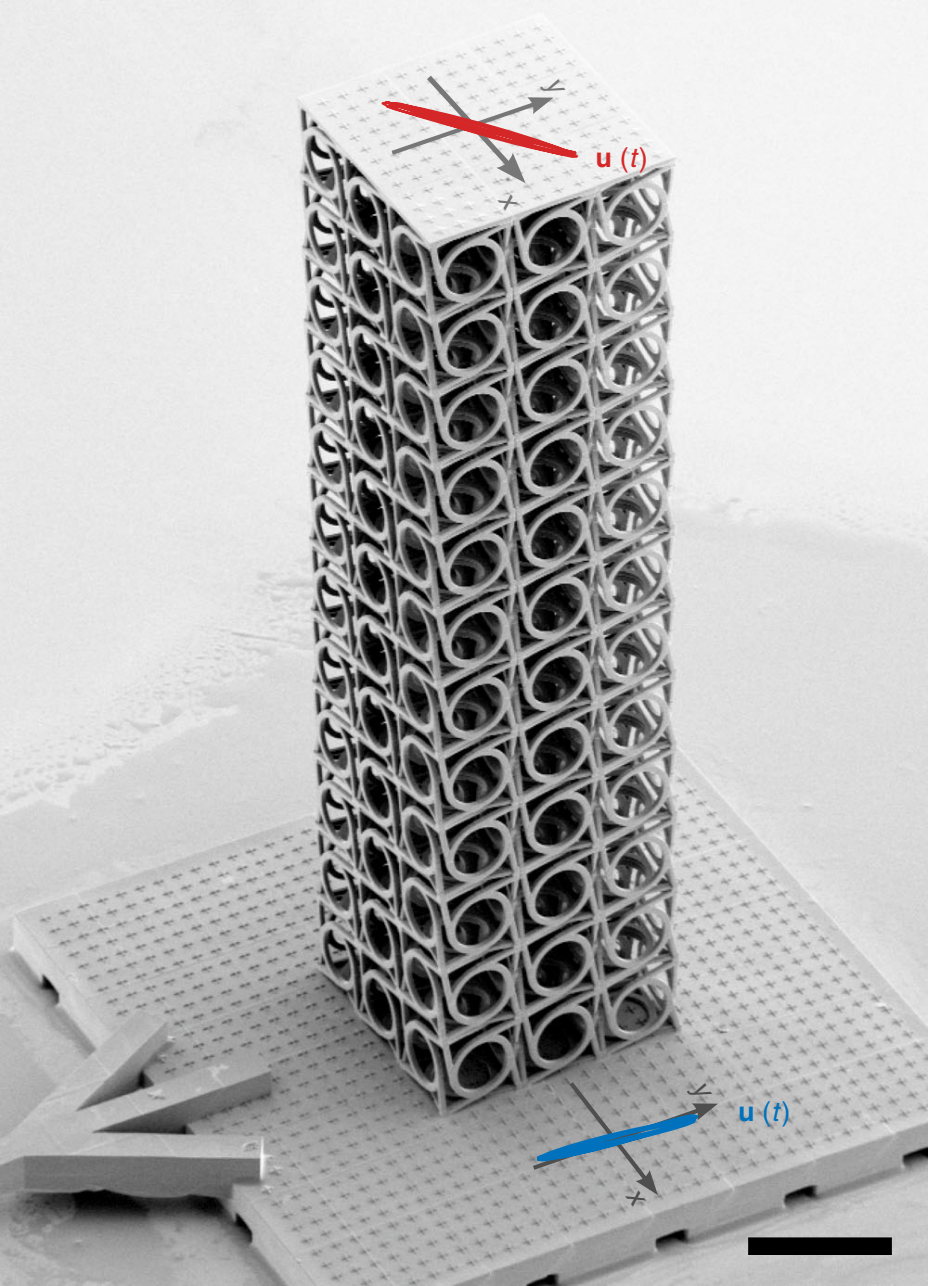
Oblique-view electron micrograph with overlaid measurement. The sample contains *N*_*x*_ = *N*_*y*_ = 3 and *N*_*z*_ = 12 unit cells (scale bar: 400 μm). A piezoelectric transducer excites the sample bottom. Top-view optical micrographs are taken under delayed stroboscopic illumination versus time delay. Displacement vectors are extracted by using image cross-correlation analysis. Results for the bottom side of the sample (blue, multiplied by a factor of 5 × 10^3^) and for the middle of the top of the sample (red, multiplied by a factor of 10^4^) are blended into the electron micrograph. For both cases, five oscillation periods are depicted to emphasize the reproducibility of the experiments (also see Supplementary Fig. 4). From these data, we derive a rotation of linear polarization due to acoustical activity of 44°. Here, the excitation frequency is 160 kHz, all other parameters are as in Figs. 1 and 2. Results from many different similar experiments on different samples are summarized in Fig. 4

We have designed our experiments based on these calculations. We fabricate the polymer samples by 3D laser nanoprinting as described previously^19^. The dedicated home-built measurement setup has not been described previously: time-harmonic transverse flexural waves are excited by a piezoelectric transducer (Physik Instrumente, PICMA Chip Actuator). The samples are directly glued onto a dedicated holder, which has been 3D printed onto the transducer (see electron micrograph in Fig. 3 and Supplementary Fig. 3). The samples are imaged from the top by a microscope objective lens (Carl Zeiss, LD Achroplan 20×, NA = 0.4). They are stroboscopically illuminated by two infrared (850 nm center wavelength) light-emitting diodes (Vishay, VSLY 3850), the current through which is pulsed with a constant duty cycle of 1.5% (e.g., corresponding to 125 ns pulse duration for a frequency of 120 kHz), and synchronized with respect to the sinusoidal piezoelectric excitation. In this fashion, we take snapshots of the displaced structure versus time. By varying the time delay between excitation and illumination, we acquire slow-motion movies of the markers on top of the plate or at the side of the bottom of the sample. The latter serve to control the incident wave polarization and behavior. Using image cross-correlation analysis^36^, we detect displacements that are much smaller than the wavelength of the light used for illumination and which are much smaller than one pixel of the complementary metal–oxide–semiconductor black/white camera (FLIR Systems BFLY-PGE-50S5M-C) used to record the images.

### Experimental data

**Fig. 4:**
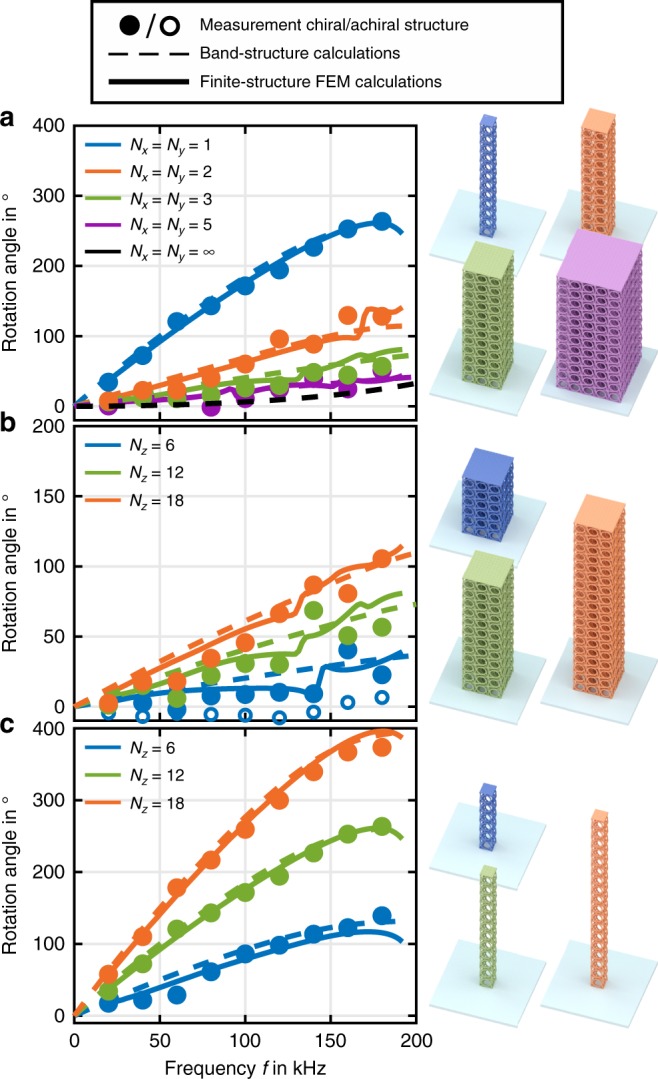
Measurements and calculations of acoustical activity. The rotation angle of an incident linear polarization upon propagation of a transverse elastic (flexural) wave is obtained from measurements (full dots) analogous to the example shown in Fig. 3 and plotted versus excitation frequency *f*. Results from band-structure calculations (cf. Fig. 2) are additionally shown as dashed curves, results from frequency-domain finite-sample calculations as solid curves (here *E* = *E*^'^ + *E*^''^ = 4.18 GPa + i0.20 GPa). **a** Rotation angle versus excitation frequency *f* for different beam cross-sections *N*_*x*_ = *N*_*y*_ and fixed beam height *N*_*z*_ = 12. **b** Same as **a**, but for fixed beam cross-section *N*_*x*_ = *N*_*y*_ = 3 and different heights *N*_*z*_. **c** As **b**, but *N*_*x*_ = *N*_*y*_ = 1. The miniatures on the right-hand side, which are colored according to the experimental data points, illustrate the parameter variations. The largest measured rotation is 22° per unit cell for *N*_*x*_ = *N*_*y*_ = 1 and *f* = *ω*/2*π* = 180 kHz. Achiral control samples show zero polarization rotation within the experimental error (see open blue dots in (**b**))

Example measurements merged into an oblique-view scanning electron micrograph of a metamaterial structure are shown in Fig. 3. The red (blue) scale bar refers to the measured displacement vectors in the middle of the top plate (at the side of the bottom of the sample). These data are shown for five consecutive oscillation periods, demonstrating the reproducibility of the measurements. In this particular example, we derive a polarization rotation of 44° (also see Supplementary Fig. 4). These and further results are summarized in Fig. 4 (full dots) and compared with results from the band-structure calculations (dashed curves) and results from frequency-domain finite-sample calculations (solid curves) already discussed above. The overall agreement between experiment and theory is very good. All qualitative trends are reproduced (as can be seen in Fig. 4a–c), the rotation angle generally increases with increasing frequency. This increasing deviation from Cauchy elasticity is expected based on our reasoning that the fixed unit cell size increases with respect to the wavelength with increasing frequency, moving away from the limit of “point mechanics”. This behavior must not be misinterpreted in terms of a resonance. It also occurs in generalized continuum theories^27–29^, which do not account for resonances. Figure 4a additionally demonstrates that the rotation angle is maximum for slender beams composed of only a single-unit cell in the cross-section (*N*_*x*_ = *N*_*y*_ = 1). It decreases toward a certain finite level versus increasing beam cross-section toward a 3D bulk metamaterial (*N*_*x*_ = *N*_*y*_ = *L*/*a* ≫ 1). The rotary power stays finite in the bulk (see dashed black curve) because the unit cell size *a* is finite compared to the wavelength *λ*, again deviating from the limit of “point mechanics”. Figure 4b, c shows that the rotation angle increases versus beam length or height *N*_*z*_, regardless of the beam cross-section (which is different in b and c). This behavior is analogous to the rotation angle in optical activity increasing proportionally with the propagation coordinate.

### Comparison with theory

The calculations for finite metamaterial samples shown by the solid curves in Fig. 4 exhibit weak wiggles superimposed on a monotonous behavior. We have traced their origin back to mass-spring wobbling resonances, with the mass given mainly by the top plate. The wobbling eigenfrequency decreases with increasing total weight of the top plate, hence it decreases with increasing beam cross-section. Due to the sample chirality, the wobbling motion induces an additional polarization rotation, which is an artifact in the sense that it does not occur in the band-structure calculations (which refer to *N*_*z*_→∞). The behavior near the wobbling resonances depends on whether damping is accounted for. In the calculations shown in Fig. 4, we have added a finite imaginary part to the polymer’s Young’s modulus according to *E* = *E*^'^ + i*E*^''^ = 4.18 GPa + i0.20 GPa. This complex value has been obtained from independent experiments on tuning forks made by the same 3D laser nanoprinting process under comparable conditions and measured at comparable excitation frequencies (see Supplementary Fig. 5). For comparison, Supplementary Fig. 6 shows computations for *E*^''^ = 0. Apart from the vicinity of the artifact resonances, the observed polarization rotation does not depend on *E*''. Intuitively, damping reduces the amplitudes of all polarization components alike; therefore, it changes neither the polarization state nor its evolution, making acoustical activity a robust phenomenon.

In conclusion, we have experimentally demonstrated the phenomenon of acoustical activity, the mechanical counterpart of optical activity. Based on generalizations of Cauchy elasticity, acoustical activity in chiral continua has previously been predicted theoretically. Our ultrasound experiments on 3D chiral mechanical metamaterials reveal linear polarization rotations as large as 22° per metamaterial unit cell. These chiral degrees of freedom provide an unprecedented control of the polarization of elastic waves in three dimensions. By scaling the metamaterial lattice constant *a*, a large range of operation frequencies is accessible.

## Methods

### Sample fabrication

We fabricate the polymer samples by 3D laser nanoprinting (Nanoscribe GmbH, Photonics Professional GT) using a commercially available photoresist (IP-S, Nanoscribe GmbH). During the printing process a 25× objective lens (numerical aperture NA = 0.8, Carl Zeiss) was dipped directly into the photoresist. Underlying 3D models were created in STL-file format using COMSOL Multiphysics (COMSOL Inc.) and further processed using Describe (Nanoscribe GmbH). Therein, the 3D models were split into lines with a horizontal distance between adjacent lines of 0.3 μm and a vertical distance of 0.7 μm between adjacent planes. The scanning of the galvanometric mirrors led to a laser focus speed of 0.1 ms^−1^, and the laser power was set to 45% of the maximum output power. To remove the unexposed photoresist, the samples were first placed in a bath of mr-Dev 600 (20 min), followed by a bath of acetone (2 min) and finally placed in a bath of isopropanol (20 min). Afterwards, the sample was ultraviolet cured for 20 min.

### Finite-element method (FEM) band-structure and eigenmode calculations

For the finite-element band-structure calculations *ω*(*k*_*z*_), we solved the eigenvalue problem using COMSOL multiphysics. We used a three-dimensional model typically meshed into a few million tetrahedra and applied second-order ansatz functions (MUMPS solver). In the case of the infinite crystal (*N*_*x*_ = *N*_*y*_ = *N*_*z*_ = ∞), the cubic unit cell as shown in Fig. 1c was implemented with Bloch-periodicity on all sides of the cube. In the cases of finite footprints (*N*_*x*_ = *N*_*y*_ = 1,2,3,5 and *N*_*z*_ = ∞) *N*_*x*_ × *N*_*y*_ × 1 unit cells were implemented. Bloch boundary conditions were only applied along the *z*-direction, whereas all other boundaries were left open (i.e., traction free). A linear elastic material was assumed with Young’s modulus *E* = 4.18 GPa, a Poisson’s ratio *ν* = 0.4, and a mass density *ρ* = 1.15 g cm^−3^. In order to isolate the structural properties of the unit cell from the ones of the constituent material (Young’s modulus, Poisson’s ratio, and mass density), the left vertical frequency scale *f* was converted to *a*/*λ*_0_ (right vertical scale). Here, *λ*_0_ is the wavelength of the constituent’s longitudinal mode, given by *c*/*f*, with *c* being the phase velocity of the longitudinal mode $c = \sqrt {\frac{{E(1 - \nu )}}{{\rho (1 - 2v)(1 + \nu )}}}$.

### Finite-structure FEM calculations

We deduced the polarization rotation angle of the finite structures containing *N*_*x*_ × *N*_*y*_ × *N*_*z*_ unit cells by solving the eigenvalue problem for a given frequency *ω* using COMSOL multiphysics. As in the experiment, a plate with the same footprint *N*_*x*_*a* × *N*_*y*_*a* as the structure and with a thickness of 10 μm was attached to the top. We prescribed a displacement at the bottom of the metamaterial structure. All other boundaries were left open (i.e., traction free). The polarization of the eigenmode was calculated by averaging the displacement field components *u*_*x*_ and *u*_*y*_ over a square with side length $\frac{3}{4} \times a$ at the center of the top plate. This averaging size is comparable to the distance between the positions of the markers tracked in the experiment. A linear elastic material was assumed with a complex-valued Young’s modulus *E* = 4.18 GPa + i0.20 GPa, a Poisson’s ratio *ν* = 0.4, and a mass density *ρ* = 1.15 g cm^−3^. The only exception are the dots in Fig. S6, where we have used *E* = 4.18 GPa (with zero imaginary part) for comparison.

## Supplementary information

Supplemental Information

Supplementary Video 1

Description of Additional Supplementary Files

## Data Availability

The data that support the findings of this study are available from the corresponding author upon reasonable request.
