# Supplementary material for: Ultrasound experiments on acoustical activity in chiral mechanical metamaterials
Source: Nat Commun. 2019 Jul 29;10:3384. doi: 10.1038/s41467-019-11366-8 (PMC6662661; doi:10.1038/s41467-019-11366-8)
Supplement: Supplementary file 1 — Supplemental Information [file 41467_2019_11366_MOESM1_ESM.pdf]

Supplementary Information:

Ultrasound Experiments on Acoustical Activity in Chiral Mechanical Metamaterials

Tobias Frenzel[1], Julian Köpfler[1,2], Erik Jung[1], Muamer Kadic[1,2,3], and Martin Wegener[1,2,*]

Martin.Wegener@kit.edu

Affiliations:

[1]Institute of Applied Physics, Karlsruhe Institute of Technology, 76128 Karlsruhe, Germany

[2]Institute of Nanotechnology, Karlsruhe Institute of Technology, 76021 Karlsruhe, Germany

[3]Institut FEMTO-ST, CNRS, Université de Bourgogne Franche-Comté, 25000 Besançon, France

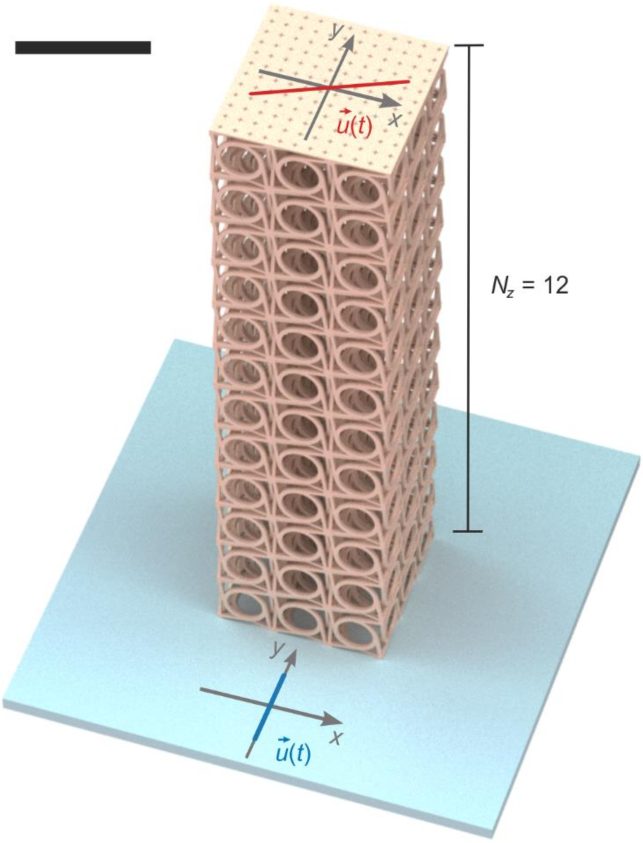

Supplementary Figure 1: Frequency-domain finite-element calculation of a finite structure.

Calculated displacement of a metamaterial beam containing $3 \times 3 \times 12$ unit cells (cf. Fig. 3) for an excitation frequency of $\omega = 2\pi \times 160\ \mathrm{kHz}$. As in the experiment, a plate with the same footprint $N_x a \times N_y a$ as the structure and with a thickness of $10\ \mu\mathrm{m}$ has been attached to the top (scale bar: $400\ \mu\mathrm{m}$). The polarization axis at the bottom is determined by the excitation. The polarization axis at the top has been calculated by averaging the displacement vector components u_x and u_y over a square with side length $\frac{3}{4} \times a$ at the plate's center. This averaging size is comparable to the distance between the positions of the markers tracked in the experiment. The results from the bottom side of the sample (blue, multiplied by a factor of $5 \cdot 10^3$) and for the middle of the top sample (red, multiplied by a factor of 10^4) are blended into the rendered metamaterial. From these date we derive a rotation of polarization due to acoustical activity of $65°$. The other parameters are as in Fig. 1.

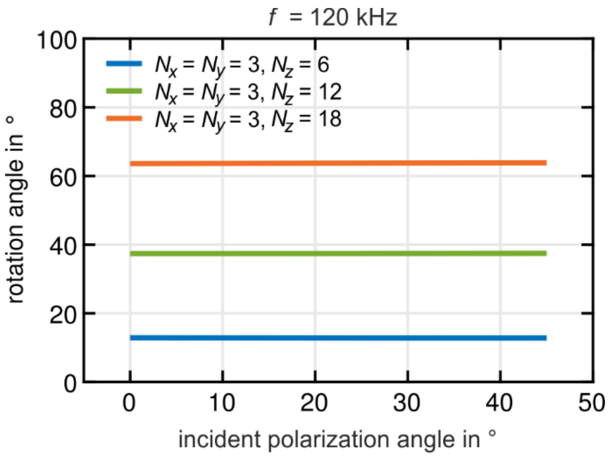

Supplementary Figure 2: Finite-element calculations for varying incident linear polarizations. Rotation angle of the linear polarization of a flexural wave upon propagation over N_z unit cells along a principal cubic axis versus the angle of the incident polarization with respect to the principle cubic axes of the unit cell, as obtained from numerical finite-element frequency-domain calculations at fixed frequency. Finite structures with $N_x = N_y = 3$ and $N_z = 6, 8$, and 12 (cf. Fig. 4b) are depicted at a fixed excitation frequency of $f = 120$ kHz. The calculations show no significant dependence of the rotation angle on the orientation of the incident linear polarization.

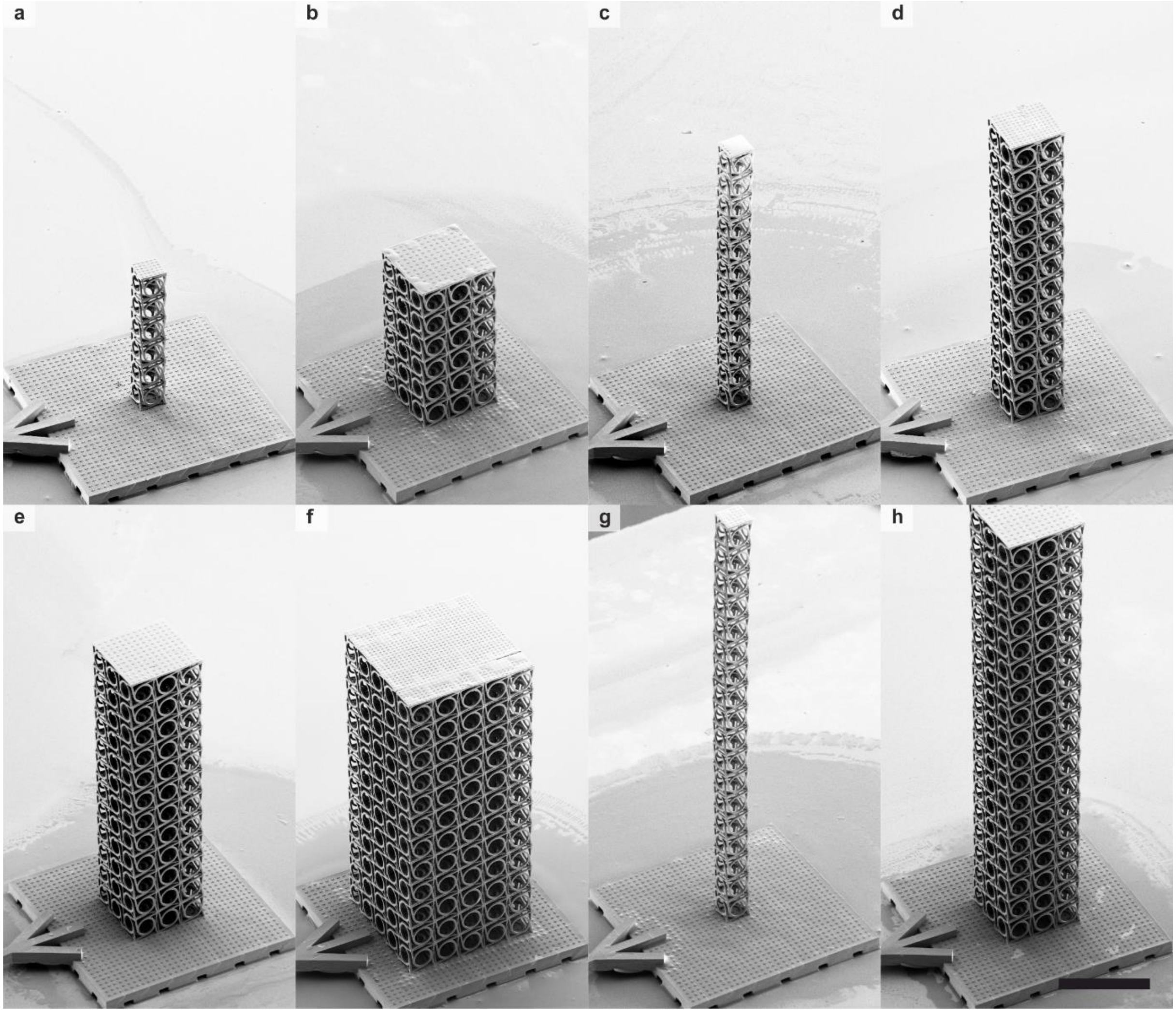

Supplementary Figure 3: Gallery of oblique-view electron micrographs of fabricated 3D chiral metamaterial beams. Samples with various aspect ratios and total number of unit cells $N_x \times N_y \times N_z$ are shown (scale bar: $800\ \mu\mathrm{m}$). Depicted beams contain (a) $1 \times 1 \times 6$, (b) $3 \times 3 \times 6$, (c) $1 \times 1 \times 12$, (d) $2 \times 2 \times 12$, (e) $3 \times 3 \times 12$, (f) $5 \times 5 \times 12$, (g) $1 \times 1 \times 18$, and (h) $3 \times 3 \times 18$ unit cells. The sample depicted in panel (e) is identical to the one shown in Fig. 3.

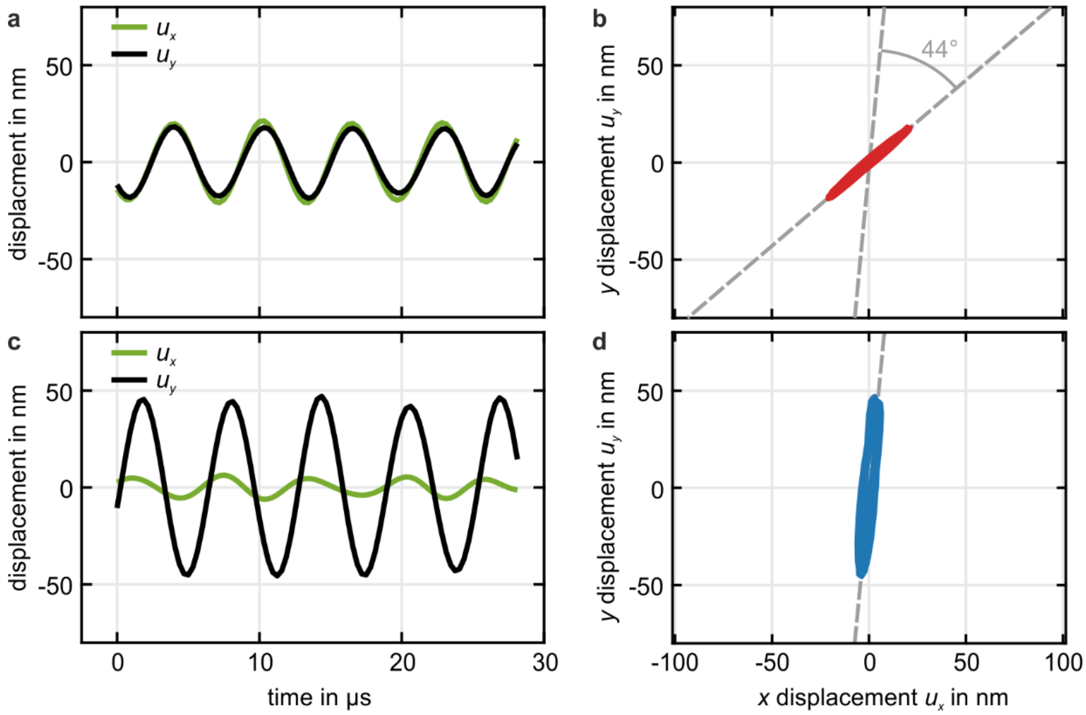

Supplementary Figure 4: **Exemplary measurement of acoustical activity.** Raw data for a structure containing $3 \times 3 \times 12$ unit cells for an excitation frequency of $f = 160$ kHz are depicted. (a) shows the extracted x- and y-components u_x and u_y of the displacement vector measured at the top of the metamaterial beam as a function of time. (b) same data as depicted in (a). The grey dashed line indicates the orientation of the linear polarization. (c) same as (a) but measured at the bottom of the sample. (d) same data as depicted in (c). In comparison with (b) the direction of the linear polarization is rotated by $44°$ after propagation through the metamaterial beam. The data from panels (b) and (d) are also depicted in Fig. 3.

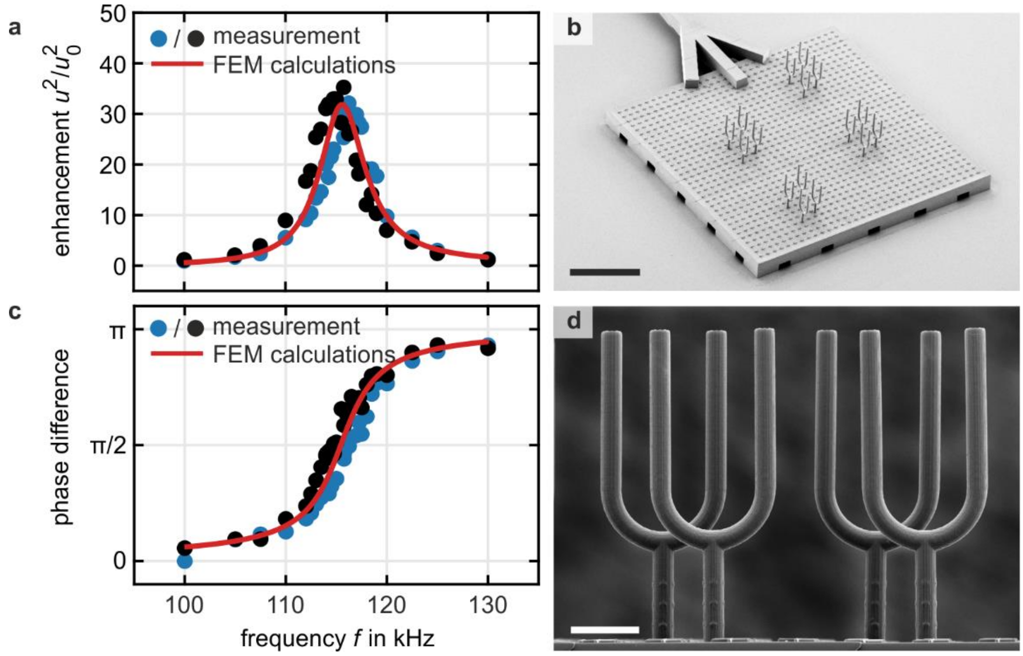

Supplementary Figure 5: Polymer tuning forks. Experiment and finite-element calculations have been performed to independently determine the real and imaginary part of the polymer's Young's modulus E in the relevant frequency range. (a) Measured enhancement u^2/u_0^2 of the amplitude versus excitation frequency for excitation with a piezoelectric transducer as in the main paper. The displacement u_0 is along the vertical direction in panel (d), measured by a second camera (cf. three markers on the tuning fork stems in panel (d)), u is the measured displacement along the horizontal direction in panel (d) at the end of the tuning fork's arms. The blue and black dots correspond to two different yet nominally identical tuning forks. The red curve corresponds to a finite-element calculation in which we have assumed a circular cross section with radius $r = 9.29\ \mu\text{m}$ throughout the entire arms of the tuning forks. For small damping, the real part of the polymer's Young's modulus predominantly determines the resonance position, the imaginary part the width of the resonance. (b) Oblique-view electron micrograph showing the holder and a set of tuning forks (scale bar: $500\ \mu\text{m}$). (c) Phase corresponding to panel (a). (d) Side-view electron micrograph of the same structure as in panel (b) (scale bar: $60\ \mu\text{m}$). From these measurements, we determine a Young's modulus of $E = 4.18\ \text{GPa} + \text{i}\ 0.20\ \text{GPa}$. We determine a statistical (systematic) error for the real

part of ± 0.07 GPa (± 0.32 GPa) and for the imaginary part of 0.01 GPa (± 0.02 GPa). The statistical error is the standard deviation obtained from two different measurements, the systematic error has been estimated on the basis of different numerical calculations with a non-constant cross section of the tuning fork arms.

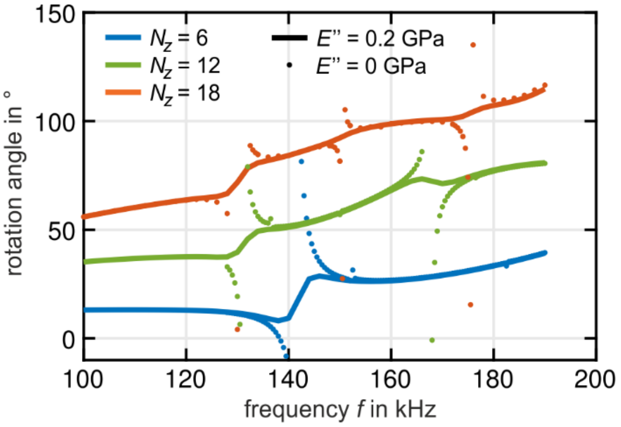

Supplementary Figure 6: Finite-element calculations for zero and finite damping. The solid curves are finite-element frequency-domain calculations of rotation angle versus frequency as for the solid curves in Fig. 4b ($3 \times 3 \times N_z$ unit cells and Young's modulus $E = E' + \mathrm{i}E'' = 4.18\ \mathrm{GPa} + \mathrm{i}\ 0.20\ \mathrm{GPa}$). The dots shown in addition correspond to the same parameters but zero imaginary part of the polymer's Young's modulus, $E'' = 0$. Without damping, the wobbling resonances discussed in the main text become more pronounced. However, apart from the vicinity of these resonances, the behavior of the rotational angle does not depend on the damping, making acoustical activity a robust phenomenon. For the other panels in Fig. 4, the wobbling resonances are less pronounced.
