# Supplementary material for: Ultrasound experiments on acoustical activity in chiral mechanical metamaterials
Source: Nat Commun. 2019 Jul 29;10:3384. doi: 10.1038/s41467-019-11366-8 (PMC6662661; doi:10.1038/s41467-019-11366-8)
Supplement: Supplementary file 3 — Description of Additional Supplementary Files [file 41467_2019_11366_MOESM3_ESM.docx]

**Title:** Supplementary Video 1
**Description:** Animation of a calculated chiral phonon eigenmode. Left: An eigenmode with eigenfrequency 𝜔1 is propagating along the 𝑧-direction in an infinitely extended 3D chiral micropolar metamaterial crystal. For clarity, all unit cells except for one column along the propagation axis (𝑧-axis) are shown semi-transparent and the displacements are largely exaggerated. Right: Simplified representation only showing the displacement vector of the unit cells’ centers of mass, forming a helix that is moving along the 𝑧-axis versus time. A snapshot is of this video is shown in Fig. 1. The wave number is 𝑘𝑧 = 𝜋/(4𝑎) and the angular frequency is 𝜔1 = 2𝜋 × 107 kHz.
